# Breeding System and Response of the Pollinator to Floral Larceny and Florivory Define the Reproductive Success in *Aerides odorata*

**DOI:** 10.3389/fpls.2021.767725

**Published:** 2022-01-14

**Authors:** Arjun Adit, Vineet Kumar Singh, Monika Koul, Rajesh Tandon

**Affiliations:** ^1^Department of Botany, University of Delhi, New Delhi, India; ^2^Department of Botany, Acharya Narendra Dev College, University of Delhi, New Delhi, India; ^3^Department of Botany, Hans Raj College, University of Delhi, New Delhi, India

**Keywords:** floral herbivory, foraging guilds, orchids, nectar robbing, mixed-mating

## Abstract

Consumption of pollination reward by felonious means in a plant species can influence the foraging behavior of its pollinator and eventually the reproductive success. So far, studies on this aspect are largely confined to interaction involving plant-pollinators and nectar robbers or thieves. However, a foraging guild in such interactions may also include floral herbivores or florivores. There is a paucity of information on the extent to which nectar larcenists may influence the foraging behavior of the pollinator and reproductive fitness of plants in the presence of a florivore. We investigated various forms of larceny in the natural populations of *Aerides odorata*, a pollinator-dependent and nectar-rewarding orchid. These populations differed in types of foraging guild, the extent of larceny (thieving/robbing), which can occur with or without florivory, and natural fruit-set pattern. The nectariferous spur of the flower serves as an organ of interest among the foraging insects. While florivory marked by excision of nectary dissuades the pollinator, nectar thieving and robbing significantly enhance visits of the pollinator and fruit-set. Experimental pollinations showed that the species is a preferential outbreeder and experiences inbreeding depression from selfing. Reproductive fitness of the orchid species varies significantly with the extent of floral larceny. Although nectar thieving or robbing is beneficial in this self-compatible species, the negative effects of florivory were stronger. Our findings suggest that net reproductive fitness in the affected plant species is determined by the overarching effect of its breeding system on the overall interacting framework of the foraging guild.

## Introduction

Floral nectar, the major pollination reward among the flowering plants, is presented to the pollinators in two ways – openly or selectively. The selective mode is associated with flowers in which nectar is concealed. The hidden floral reward can be consumed by a suitable pollinator only when it is legitimately accessed (Fenster, 1991). The dynamics of production and presentation of nectar by a plant play a crucial role in maintaining constancy with suitable pollinators and sustaining fruit-set (fitness). Altered foraging behavior of the pollinator can adversely influence the fitness when there is discontinuous provisioning of rewards. A variety of foragers who illegitimately access the reward meant for pollination service (floral antagonists) are known to cause an aberrant resource availability by integrating into plant-pollinator interaction (Irwin et al., 2001, 2010). The adverse consequences on plant fitness become more pronounced when the rewards are not replenished and force the pollinator to leave or shift to a better resource in the community.

In general, plant taxa that usually bear nectar spurs or tubular corolla (such as in Bignoniaceae, Ericaceae, Fabaceae, and Lamiaceae) may experience some or other form of larceny or florivory (Irwin and Maloof, 2002). In terms of the extent, larceny may range from thieving to the robbing of pollination rewards (Inouye, 1980). It refers to thieving when reward collection is made through a legitimate route without pollination success and it is termed robbing when the access is gained by piercing the corolla/calyx (Irwin et al., 2010). However, when a forager mutilates a flower or consumes floral parts, the visits fall in the realm of florivory or floral herbivory (McCall and Irwin, 2006; Oguro and Sakai, 2009). At any given time, a plant species may serve as a floral resource for all such antagonistic foragers besides the pollinator (foraging guild).

Nectar larcenists are known to affect the behavior of pollinators to different extents, resulting in varied outcomes on plant fitness. These outcomes may range from detrimental to partial-negative, weak-positive, or beneficial (Morris, 1996; Traveset et al., 1998; Maloof and Inouye, 2000; Navarro, 2000; Singh et al., 2014; Zhang et al., 2014). So far, the consequences on plants have been evaluated from interactions that are mainly comprised of plant-pollinator-robbers in a foraging guild. It has been shown that the robbers may either directly influence the fitness of plants or do so indirectly, by altering the behavior of pollinators (Hazlehurst and Karubian, 2016). These outcomes may depend on a variety of other factors, such as the mating system of the plant species, community assemblages, and differences in geographic regions (Barrett and Harder, 1996; Pellissier et al., 2012; Stanton-Geddes et al., 2012). However, there is a paucity of information on the extent to which the consequence may vary within a species in its distribution range, especially when the foraging guilds are represented by more than one type of floral antagonists. A few studies involving florivory and robbing within a species have shown that florivores avoid direct conflict with robbers by foraging at different stages of flower development (*Arctostaphylos pungens*; Eliyahu et al., 2015), or the pollinator starts behaving as a robber in the event of florivory (*Iris bulleyana*; Ye et al., 2017). In such interactions, florivory incurs a direct cost on pollination service (Krupnick et al., 1999).

Orchids are known to exhibit diverse floral forms and attract a variety of foragers through specialized cues (Fay and Chase, 2009). Yet, the consequences of floral antagonists on the plant-pollinator interaction have been insufficiently explored among orchids. In the present study on nectar rewarding *Aerides odorata* (*A. odorata*), an orchid species with antagonists (nectar robber, thief, and florivore) in its foraging guild, we investigated the (i) breeding system of the species, (ii) foraging behavior of various types of insects that depend on the flower as resource, (iii) influence of the antagonists on the pollinator in the foraging guild, and (iv) impact of altered behavior of the pollinator on the reproductive fitness of plants. Our study suggests that floral larceny in pollinator-dependent autogamous species would yield positive effects through enhanced pollination success, provided that the pollinator is not discouraged from visiting the flowers.

## Materials and Methods

### Study Species

**FIGURE 1:**
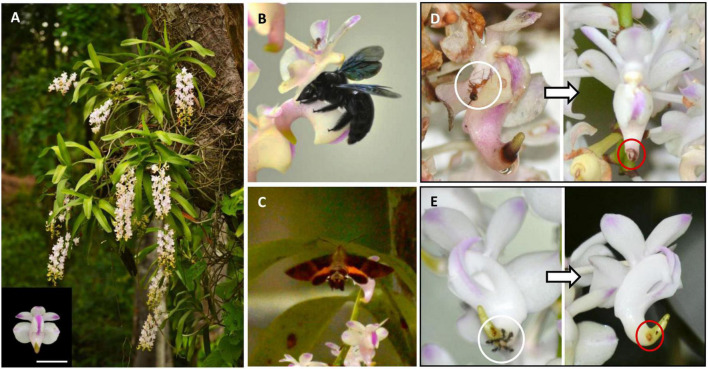
Floral visitors in *Aerides odorata*. **(A)** A flowering patch near Tlaksih Village. The flowers are arranged in pendulous racemes. Inset: A freshly opened flower with shiny-green forward facing hooked spur (scale = 1 cm). **(B)** *Xylocopa nasalis* pollinating the flower. **(C)** *Macroglossum belis* thieving on the flower. **(D)** The florivore, *Anoplolepis gracilipes* (white circle), is involved in the removal of the spurred nectary tip (red circle), **(E)** *Paratrechina* sp. (white circle) acts as a robber by piercing the nectary (red circle).

We selected *A. odorata* Lour. (Orchidaceae), commonly known as “Cat’s-tail Orchid” for this study, which is native to south and south-east Asia (Plants Of the World Online, 2021). This epiphytic orchid is monopodial and grows luxuriantly in open forest patches with access to abundant sunlight. The flowers develop on drooping axillary racemes and emit a strong fruity fragrance. Each raceme has 35–40 spirally arranged flowers that open acropetally. As in other species of *Aerides*, flowers of *A*. *odorata* have a forward facing and hooked spur of petal origin (Figure 1A). A voucher specimen of the orchid has been submitted to the Delhi University Herbarium, University of Delhi, New Delhi, India (*DUH 14670*).

### Populations Investigated

The study was carried out in three natural populations located in the state of Tripura, north-east India (Table 1). Among these, Clouded Leopard National Park (CLNP) is a part of the protected forest, and the remaining two (Kumarghat town; KT and Tlaksih village; TV) are located at the outskirts of inhabited areas. The plants in all the populations showed peak flowering by mid-June. We identified these three populations on the basis of the type of floral foraging guild. Our analysis during the preliminary survey (2017) showed that these populations also varied in the extent of natural fruit-set pattern (range 10–65%), which indicated the influence of foraging guilds on fitness of the plants. The consistency of this pattern in natural fruit-set was ascertained quantitatively for every flowering season during the study period (2018–2020). For this, 10 flowers on randomly selected 20 plants were tagged during each season and in each population.

**TABLE 1:** Location, foraging guilds, and natural fruit-set pattern in the three study populations.

Study population	Geo-coordinates	Composition of foraging guild	Natural fruit-set (%)		
			2018	2019	2020
Clouded Leopard National Park (CLNP)	23.675°N, 91.320°E, 47 m	Pollinator, Thief, and Robber	65	60	62
Tlaksih village (TV)	24.032°N, 92.278°E, 630 m	Pollinator	31	29	32
Kumarghat town (KT)	24.182°N, 92.041°E, 24 m	Pollinator, Thief, and Florivore	11	10	11

*Percent fruit-set was ascertained from a set of randomly tagged 200 flowers in each population during each flowering season.*

### Floral Biology

#### Flowering Phenology

Flowering phenology was recorded from a set of 100 random plants in each population. The plants were monitored by making regular weekly visits during the first season of flowering and fruiting. Floral longevity was ascertained by recording observations on flowers (*n* = 5 racemes) from five randomly selected plants in each population; the racemes were bagged with nylon mesh to prevent visits of the foragers.

#### Floral Reward

The average volume of nectar produced by a freshly opened flower (*n* = 20 flowers from three populations) was quantified using Single Channel Micropipette (Corning Inc., Corning, NY, United States, 2–20 μl Lambda). To ascertain nectar replenishment, nectar was manually removed from the same set of flowers; the flowers were monitored at regular intervals. Sucrose concentration as sugar equivalent was estimated using a temperature compensated hand-held refractometer (Erma Inc., Tokyo, Japan, number 11-520-0). Nectar was also analyzed qualitatively for the presence of phenolics, alkaloids, and proteins by the colorimetric method. For this, pointed end of the triangular paper strip, prepared from Whatman number 1 filter paper, was inserted into the excised flower spur to load nectar. The strips were air dried and tested for the presence of phenolics with Folin-Ciocalteu’s reagent, alkaloids with Dragendorff’s reagent, and proteins with bromophenol blue (Dafni et al., 2005).

### Breeding System

The breeding system of *A*. *odorata* was studied through experimental pollinations, after ascertaining the receptive duration of the stigmatic cavity with the peroxidase test (Shivanna and Tandon, 2014). To gather a population perspective, the exercise was carried out for three seasons on randomly selected plants in each population. The selected plants were used for all the treatments, and the set of treated plants differed every season in each population. For each treatment, 70 flowers (*n* = 14 plants) from CLNP, 60 flowers (*n* = 12 plants) from TV, 30 flowers (*n* = 6 plants) from KT were treated during each season of the study period. The treatments included (i) apomixis: pollinia were removed and flowers were bagged; (ii) spontaneous autogamy: flowers were bagged without causing any disturbance; (iii) facilitated autogamy (FA): for pollination, the pollinia were sourced from different flowers of the same inflorescence; and (iv) Xenogamy: pollinia were taken from a flower on a different plant. The treated flowers were bagged after pollination and monitored for fruit-set.

Inbreeding depression, ID, (δ_t_), was computed as δ_t_ = 1 – (ω_s_/ω_o_), where ω_s_ and ω_o_ refer to fruit-set in self-pollinated and cross-pollinated flowers, respectively (Charlesworth and Charlesworth, 1987). The negative value indicates outbreeding depression while the positive value corresponds to ID.

### Pollination Ecology

#### Identity of Floral Visitors and Foraging Guilds

Observations were made during the peak flowering period to record the floral visitors, their time of visits on the flower, and foraging behavior. The visitors were categorized into four types on the basis of their foraging behavior. (1) *Pollinator*: foragers which accessed the nectar through the legitimate opening of flowers and carried pollinaria on their body. All those visitors who accessed nectar without resulting in pollination were considered as nectar larcenists (Inouye, 1980), namely (2) *Thief*: foragers who accessed nectar through the legitimate opening, (3) *Robber*: visitors who accessed nectar by piercing the spur, and (4) *Florivore*: visitors who excised the tip of the nectariferous spur. These foragers were represented in different combinations at three study sites (Table 1).

#### Effect of Larceny and Florivory on Pollinator Behavior and Fruit-Set

We recorded the foraging behavior of the pollinator and the thief in terms of their flower-handling time and foraging frequency. Foraging frequency was determined as the number of flowers visited on the tagged flowers (*n* = 10 bouts in each population). Flower-handling time was monitored by using a digital stopwatch (*n* = 20 and *n* = 25 tagged flowers per population for the pollinator and thief, respectively).

The effect of larceny (thieving and robbing) and florivory was determined by tagging the flowers that were thieved (*n* = 25, at KT and CLNP, each season), robbed (*n* = 250, at CLNP), or mutilated (*n* = 250, at KT). The values were expressed as a percentage. The data were recorded during the peak time of two flowering seasons (2019 and 2020) and plotted along with the observed natural fruit-set from the three populations.

### Statistical Analyses

All data were verified for normal distribution with the Shapiro-Wilk test; homogeneity of variance with Levene’s test; and pairwise comparisons with *post hoc* Tukey honestly significant difference (HSD). Percentage data were root-square arcsine transformed to achieve homoscedasticity (Sokal and Rohlf, 2012).

To determine whether the pollination treatments that yielded fruits [FA; and xenogamy (X)] differed significantly, one-way ANOVA was performed. The season was kept as a fixed factor and fruit-set as the dependent variable. As fruit-set did not differ by type of pollination treatments among populations in a season {[FA: *F*_(2, 8)_ = 0.62, *p* > 0.5]; and [X: *F*_(2, 8)_ = 0.8, *p* > 0.5]; One-way ANOVA}, the data for these treatments were pooled by populations. For the analysis, treatments were used as a fixed factor and fruit-set in different populations was used as a dependent variable.

The difference in foraging behavior of the pollinator and the thief was determined by performing One-way-ANOVA. The populations were considered as a fixed factor; and flower handling time and foraging frequency were considered as the dependent variables.

The data were visualized using the *ggplot2* package (Wickham, 2016) in R ver. 4.0.3 (R Core Team, 2020).

## Results

### Floral Biology

#### Phenology

In *A*. *odorata*, the racemes appeared by the fourth week of April, and the floral buds are formed by the third/fourth week of May. The populations located at CLNP and KT exhibited a peak in flowering during the first week of June, while flowering in TV peaked by mid-June. After anthesis, the unpollinated flowers remained fresh and receptive for up to 18 days. The stigmatic cavity remained receptive from the day of anthesis until pollination is achieved.

#### Floral Reward

Nectar is produced and accumulated deep in the hooked region of the spur; the latter is hard and shiny-green toward the outside. Nectar production started around 0500 h and was replenished 24 h after it is removed, until the flower remained fresh or unpollinated. On average, a virgin flower had 18.98 ± 0.37 μl nectar (*n* = 20). Nectar contained 62 ± 0.72% (*n* = 10) sucrose and was tested positive for proteins; however, phenolics and alkaloids were absent.

### Breeding System

Pollination treatments revealed that the species is self-compatible. Flowers bagged to ascertain apomixis and autonomous self-pollination did not set fruit. Fruit-set from xenogamous pollination treatment (78.6%) was significantly higher [*F*_(1_,_5)_ = 5,448.94, *p* < 0.001; One-way ANOVA] than that obtained from facilitated selfing (24.1%), suggesting outcrossing nature of the species (Table 2). Open-pollination (Table 1) was highest [*F*_(2_,_8)_ = 678.70, *p* < 0.001; One-way ANOVA] at CLNP (62.3%); followed by TV (30.6%) and KT (10.6%; Tukey HSD, *p* < 0.001). Based on the outcomes of pollination treatments, the species showed a higher incidence of ID (0.69).

**TABLE 2:** Outcome (% fruit-set) of various experimental pollination treatments performed in *Aerides odorata*.

Pollination treatment	Fruit-set (%)		
	2018	2019	2020
Apomixis	0	0	0
Spontaneous autogamy	0	0	0
Facilitated selfing	25	24.37	23.12
Xenogamy	78.75	78.12	79.37

*For each treatment in every season 160 flowers (n = 32 plants) were used. As the populations did not differ by treatment (p > 0.5), the data were pooled.*

### Pollination Ecology

#### Identity of Floral Visitors and Foraging Guilds

The flowers of *A. odorata* were exclusively and legitimately pollinated by *Xylocopa nasalis* (*X. nasalis*; large carpenter bee) at all three populations between 0745 and 0830 h (Figure 1B). The bee approached the inflorescence in a zig-zag manner, before landing onto the spurred labellum. The mean flower-handling time of *X. nasalis* was 13.14 ± 2.65 s (*n* = 60 floral visits), and the foraging frequency (flowers per bout) was 54.42 ± 6.81 (*n* = 30 bouts). The bee often carried more than one pollinarium. The anther cap attached along with the pollinia caused hindrance while accessing the reward, especially when more than two pollinaria were lodged onto the thoracic region. In such instances, the bee used its legs to actively remove the anther cap to gain entry into the flower. Active removal of anther cap sometimes resulted in the loss of pollen sacs.

Among the antagonists, *Macroglossum belis* (*M. belis*; Common hummingbird hawkmoth) was involved in the thieving of rewards in two of the populations (CLNP and KT; Table 1 and Figure 1C). The moth confined its visits between 0530 h and 0630 h; they spent 8.19 ± 0.78 s (*n* = 50 floral visits) on a flower with a foraging frequency of 4.05 ± 1.23 (*n* = 20 bouts). *Anoplolepis gracilipes* (*A. gracilipes*; Crazy yellow ant; at KT; Table 1) acted as florivore, as it invariably severed the nectariferous tip of the spur while foraging (Figure 1D). Contrary to this, *Paratrechina* sp. (Crazy black ant; as seen in CLNP; Table 1) acted like a robber, because it accessed the reward by making a small hole in the nectary spur (Figure 1E). Both these ants begin their activities on the flowers before the arrival of the pollinator and remain active for the rest of the day. As the foraging guild at TV population was represented by the pollinator alone, it was used as a control to compare the effects of larceny.

#### Effect of Larceny and Florivory on Pollinator Behavior and Fruit-Set

**FIGURE 2:**
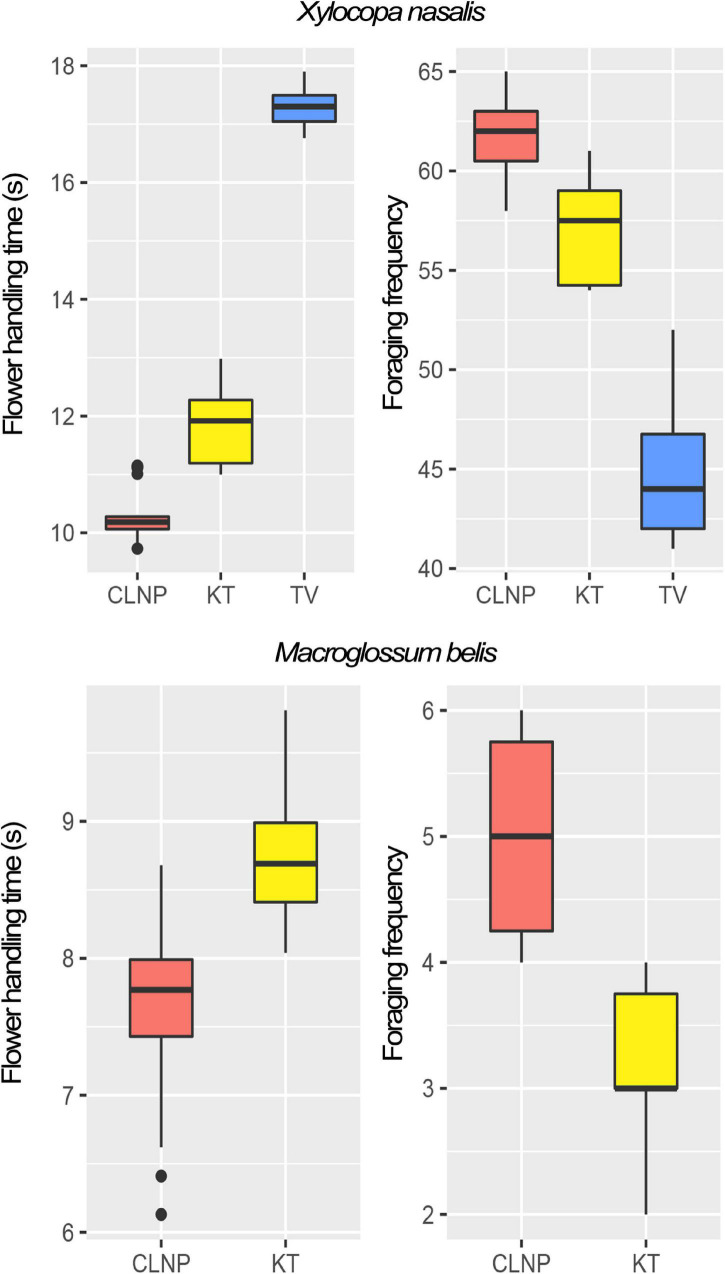
Box-plots depicting the foraging behavior of the pollinator (*Xylocopa nasalis*) and the thief (*Macroglossum belis*), in terms of flowering handling-time (s) and foraging frequency (flowers visited in a bout) in the study populations (CLNP in red; KT in yellow; and TV in blue). At TV, the thief was absent from the foraging guild. CLNP, Clouded Leopard National Park; KT, Kumarghat town; TV, Tlaksih village.

Both flower-handling time and foraging frequency of the pollinator and thief varied among respective populations (Figure 2). The flowers damaged by *A. gracilipes* (at KT) were neither visited by the pollinator (*X. nasalis*) nor by the thief (*M. belis*) and thus remained unpollinated. At CLNP, the robbed flowers (by *Paratrechina* sp.) did not deter the visits of the pollinator and the thief.

**FIGURE 3:**
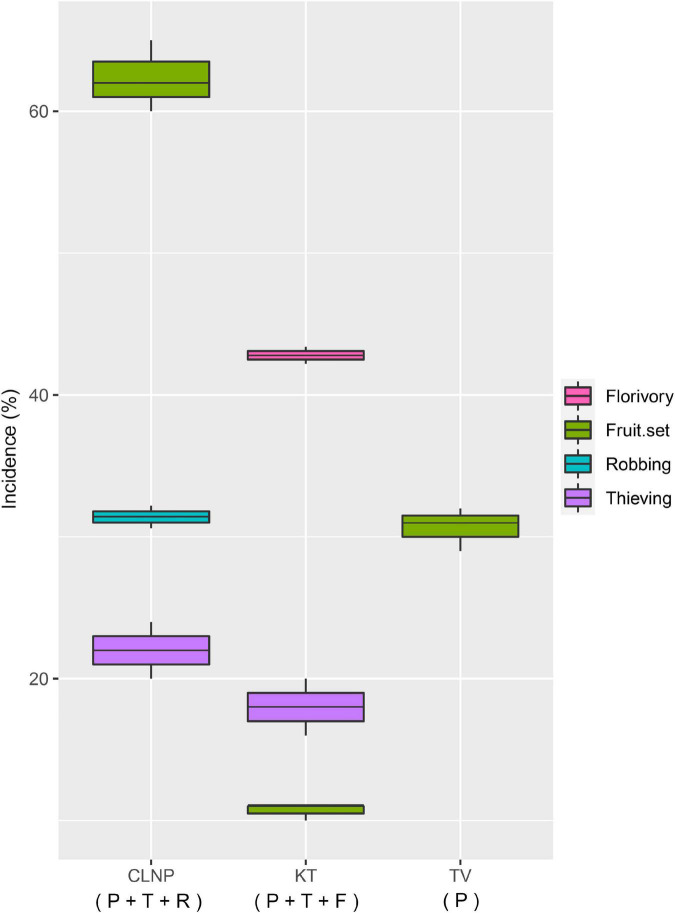
Incidence of thieving and robbing, florivory, and natural fruit-set, expressed as a percentage in different populations of *A. odorata.* P, pollinator; T, thief; R, robber; F, florivore.

The pollinator spent significantly lesser [*F*_(2_,_57)_ = 1,105.41, *p* < 0.001; One-way ANOVA] amount of time at KT (11.8 ± 0.6 s) than that at TV (17.2 ± 0.3 s). In contrast, foraging frequency of the pollinator was significantly greater [*F*_(2_,_27)_ = 91.15, *p* < 0.001; One-way ANOVA] at KT (57.1 ± 2.6 flowers per bout) than that at TV (44.9 ± 3.6 flowers per bout). There was a significant difference in the flower-handling time [*F*_(1_,_48)_ = 53.16, *p* < 0.001; One-way ANOVA] and foraging frequency [*F*_(1_,_18)_ = 29.81, *p* < 0.001; One-way ANOVA] of the thief at CLNP and KT (Figure 2). Floral damage was higher at KT (florivory, 42.8%), than that at CLNP (by robbing, 31.4%). Thieving was higher at CLNP (22%), as compared to KT (18%; Figure 3).

## Discussion

*Aerides odorata*, a nectar rewarding orchid with spurred flowers, interacts with a variety of insects that includes floral antagonists (nectar thief, robber, and florivore) besides the pollinator. The present work demonstrates that the species is self-compatible. There are varied outcomes of different forms of antagonistic interactions on the reproductive fitness (fruit-set) in this orchid. Thieving and robbing do not dissuade the pollinator from foraging the affected flowers but florivory does. Reproductive fitness of the plant is influenced positively in the presence of thieving and robbing, while florivory inflicts a negative effect.

### Breeding System

**FIGURE 4:**
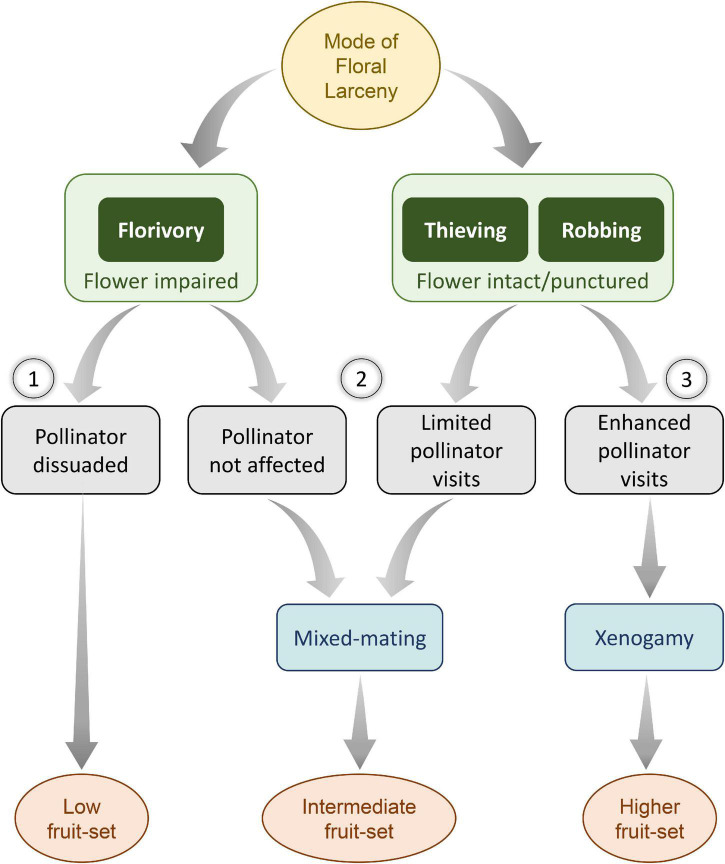
A consequence of floral larceny on reproductive fitness among preferentially outbreeding plant taxa. In such taxa, fitness is largely driven by foraging guilds, the nature and extent of larceny, and the breeding strategy adopted by the plant. When a floral organ of functional significance is excised and pollinators are dissuaded, the fruit-set is likely to decline (1). In contrast, the plants may exercise a mixed-mating strategy to result in intermediate fruit-set either due to unaltered pollinator’s behavior or limited visits of pollinators (2). Alternatively, when the organ is intact (as seen during robbing or thieving), higher than usual fruit-set may be observed through increased pollinator visits in cross-pollinated plants (3).

Experimental pollination treatments showed that *A*. *odorata* is a self-compatible species, and the pollinator appeared to be essential for facilitating autogamy. The species is a preferential outbreeder, as the FA resulted in a significantly lower amount of fruit-set. Mass floral display through compact racemes and selective reward presentation through spur also conforms to an outcrossing strategy (Harder et al., 2001). Further, lower fruit-set through autogamy indicates an early-acting ID in the species (Wallace, 2003). It is inferred that in such preferential outbreeding plants, robbing and thieving would lead to mixed-mating via geitonogamy and xenogamy, resulting in higher reproductive success (Husband and Schemske, 1996). On the other hand, florivory is most likely to dissuade the pollinators (Figure 4).

### Foraging Behavior of Insects in the Foraging Guild

The foragers exhibited diverse nectar extraction techniques in *A*. *odorata*, which led to site-specific consequences of interaction (Herrera, 1995; Valdovinos et al., 2016). Usually, carpenter bees are known to act as nectar robbers and mutilate flowers in many plant species (Irwin et al., 2010; Varma and Sinu, 2018). However, in the present study, *X. nasalis* was invariably legit in its approach and thus acted as a pollinator in all three populations. This shift in behavior is known to be governed by species-specific floral traits and community assemblages (Waser, 1983; Sargent and Ackerly, 2008). In *A. odorata*, the bee behaved as the pollinator, despite being amenable to larceny. This behavior appears to be imposed by the floral architecture, as the bee is able to access nectar only when the spur is pushed down after proper landing. Nectar analysis is also consistent with bee pollination systems. Alkaloids usually deter lepidopterans, therefore their absence in nectar may be a reason for visitation by the moth in *A*. *odorata* (Kearns and Inouye, 1993).

The efficiency of nectar removal is known to differ among the foragers. It is believed to be an inherited trait in thieves/robbers while it happens through learning among pollinators (Olesen, 1996; Zhang et al., 2007; Irwin et al., 2010). In natural habitats, ants rarely behave as pollinators and are more commonly seen playing the role of insect deterrent or robber (both primary and secondary) during interactions with plants (Dutton and Frederickson, 2012; Boaventura et al., 2021). Inflicting a damage to flowers by excising the nectary altogether could be a beneficial mode of foraging for ants at KT, as it ensures minimal loss of nectar, and the spur tips may be utilized as food by ants. The differential foraging behavior of the two species of ants can be related to the respective shape of the mandibles (Camargo et al., 2015). In *Paratrechina* sp., the mandibles are sharp and pointed and thus conducive for piercing, while that of *A. gracilipes* are short and serrated and appear to be more suited for leaf-cutting (Sinu et al., 2018; AntWeb, 2021; GISD, 2021). In contrast, moths are functionally diverse and are known to pollinate, thieve, or rob. Due to their long and thin proboscis, moths are capable of thieving and opportunistically forage on tubular and spurred flowers with minimal (none to low) floral damage (Willmer, 2011).

### Influence of Floral Antagonists on the Pollinator Behavior

In *A. odorata*, floral larceny (robbing and thieving) leads to a decrease in nectar volume in affected plants. Due to reduced nectar volume, the pollinator spends the lesser amount of time on the affected flower. Consequently, the pollinator is forced to visit a greater number of flowers in the population to fulfill its energy requirement. In doing so, pollen flow among the unrelated conspecifics is also enhanced. In contrast, florivory dissuades the pollinator to visit the mutilated flowers altogether; such flowers are also not visited by the thief. Thus, loss of the nectary directly impacts the reproductive output of the plants.

The ability to replenish nectar and the manner in which it can be accessed by the foragers are important functional aspects of a flower (Irwin et al., 2010). Failure to replenish nectar and selective accessibility to the reward can substantially influence the behavior of the foragers. Sustained nectar production or replenishment in partly damaged flowers can ensure the continuation of the visits by the pollinator. In the event of damage to the nectar secreting region, the visits of the forager can be adversely affected. However, pollinator visits would persist in a flowering (resource) patch if nectar replenishment rate is higher or the patch size is larger (Irwin et al., 2015). In *A*. *odorata*, the standing crop of nectar in all three populations did not appear to be an issue for the pollinator to leave the flowering patch. Despite a slow rate of nectar replenishment (≈24 h), the nectar pool is maintained by the presence of numerous flowers (≈40) in a raceme that opened at regular intervals (3 or 4 flowers each day). Thus, although the plant may expend more energy in nectar renewal, the cost seems to be balanced out by continuous engagement of pollinators through prolonged flowering (Newman and Thomson, 2005). However, the cost of damage appeared to be irreversible once the florivore excised the nectary through florivory. On the other hand, thieving or robbing did not affect the functionality of flower in terms of pollinator attraction.

### Impact of Altered Behavior of the Pollinator on Reproductive Fitness of the Plants

The net fitness of plants affected by larceny can be analyzed from the cumulative effect of antagonistic interactions. In our study, the population affected by robbing (CLNP) led to intense foraging by the pollinator. Thieving at both the locations (KT and CLNP) also declined the quantity of nectar. The 2-fold greater fruit-set at CLNP (robbing + thieving) correlates with increased xenogamy and shorter flower-handling time. Contrastingly, florivory reduces the original pool of virgin flowers for the pollinators by signaling the non-availability of rewards. It is opined that the repercussions of larceny can determine the eco-evolutionary course of not just the plant system, but all partners interacting in the network (Irwin and Maloof, 2002). By avoiding the reward-less flowers, pollinators can reduce the overall cost incurred on foraging (Irwin and Brody, 1998; Irwin et al., 2010). In *A*. *odorata*, the negative effects from florivory were stronger than the positive influence resulting from thieving and robbing. Thus, nectar appears to play a crucial role as a decisive cue of attraction in this species, while fragrance is the exploratory one to locate resource patches.

## Conclusion

Our study shows that the presence of florivores can transform the beneficial effects of thieving and robbing into detrimental one in the same plant species. Florivory dissuades not only the pollinator but also the other foragers in the guild. Despite being a self-compatible species, nectar robbing is beneficial for the plant. Nectar robbing and thieving impart positive influence on the fitness of the plant by altering foraging bouts of the legitimate pollinator, while florivory is responsible for reduced plant fitness. As *X. nasalis* is a solitary bee, and only a few individuals manage a population in bloom, the fitness of both the orchid and the pollinator is significantly dependent on the effects emanating from antagonistic interactions. Since robbing and thieving do not appear to adversely affect the fitness of *A*. *odorata*, and both the interacting partners are benefited, insects involved in the act qualify as “partial mutualists” rather than antagonists. Therefore, we propose that designating the role of foragers in a guild is contextual, as fitness of plants is also driven by the breeding system and the manner in which foragers interact in the network.

## Data Availability Statement

The original contributions presented in the study are included in the article, further inquiries can be directed to the corresponding author.

## Author Contributions

AA, VS, and RT designed the study. AA gathered field observations and conducted the experiments. All authors were involved in preparation of the manuscript and have approved the final version.

## Conflict of Interest

The authors declare that the research was conducted in the absence of any commercial or financial relationships that could be construed as a potential conflict of interest.

## Publisher’s Note

All claims expressed in this article are solely those of the authors and do not necessarily represent those of their affiliated organizations, or those of the publisher, the editors and the reviewers. Any product that may be evaluated in this article, or claim that may be made by its manufacturer, is not guaranteed or endorsed by the publisher.
